# Perceived barriers to eye care use among secondary school students with pinhole-improvable reduced visual acuity in two schools in Kampala, Uganda: a cross-sectional study

**DOI:** 10.1080/07853890.2026.2743336

**Published:** 2026-10-05

**Authors:** Nicholas Musisi Kagumba, Gilbert Ssebuliba, Anguyo Dralega, Naomi Nsubuga, Jemini Patel, Uday Kumar Addepalli

**Affiliations:** ^a^ Department of Allied Health Sciences, College of Health Sciences, Makerere University, Kampala, Uganda; ^b^ Eye Department, Mengo Hospital, Kampala, Uganda

**Keywords:** Uncorrected refractive errors, barriers, students, refractive services, secondary schools

## Abstract

**Introduction:**

Uncorrected refractive error (URE) is a primary cause of visual impairment (VI). Although URE causes VI and increases the risk of amblyopia in school-going children, no studies have examined barriers to uptake of refractive eye-care services among secondary school students in Uganda. This study reports the perceived reasons for the poor uptake of refractive eye care services among students at two secondary schools in Kampala, Uganda.

**Methods:**

A cross-sectional study was conducted in two secondary schools in Kampala between March 2019 and April 2019. Students’ presenting distance visual acuities (DVA) were measured. Participants with a DVA worse than 6/9 were reassessed using a pinhole. Those not using spectacles or contact lenses who had pinhole-improvable reduced distance visual acuity suggestive of uncorrected refractive error were administered a structured questionnaire. Descriptive statistics were used to analyze the data. Chi-square tests were performed to assess the association between sex and the proportion of screened students with pinhole-improvable, reduced distance visual acuity suggestive of uncorrected refractive error.

**Results:**

407 students were screened for refractive errors. In total, 10.81% (44/407, 95% CI:8.15% to 14.20%) of screened students had pinhole-improvable reduced distance visual acuity suggestive of uncorrected refractive error (URE). Perceived barriers towards refractive eye-care use reported included fear of surgery (52.27%, 95% CI: 37.94% to 66.25%), unawareness of the available eye care services (50.00%, 95% CI: 35.83% to 64.17%) and not feeling comfortable with the indigenous practitioner to whom they had access (19/44,43.18%, 95% CI: 29.68% to 57.78%). Financial limitations were stated as an important barrier by 20.00% (5/25, 20.00%, 95% CI:95% CI: 8.86% to 39.13%) of participants who stated the most important barrier.

**Conclusion:**

Among students who participated in screening at two secondary schools, a small subgroup had pinhole-improvable reduced distance visual acuity suggestive of an uncorrected refractive component. Exploratory questionnaire responses identified possible barriers related to awareness, perceptions of treatment, access, cost, and family or social influences. These findings require confirmation using refraction-based case definitions, probability sampling, and a culturally adapted and validated adolescent questionnaire.

## Introduction

Uncorrected refractive error (URE) is the second leading cause of visual impairment after cataract. By 2020, URE caused blindness in 3.7 million people and moderate or severe visual impairment in 157 million people globally [1]. Both URE and cataract are avoidable and treatable with cost-effective interventions [2]. The use of spectacles is one of the most effective ways of correcting refractive errors [3]. It is estimated that 17.43% of school-going children have refractive errors globally. The prevalence of refractive errors among school-going children in urban areas (22.7%) is much higher than that among children from rural areas (13.1%) [4].

The proportion of URE among high school students in South Africa is approximately 27% [5]. The prevalence of refractive errors among primary school-going children in Kampala is 11.6%, and 26.4% among university students [6,7]. The risk factors for refractive errors include near work (including reading and computer use), ethnicity, age, education, genetics, family history, prematurity, and diet [8].

In Uganda, pediatric eye care has faced notable disruptions, particularly the rising burden of uncorrected refractive errors [9]. URE increases the risk of amblyopia in school-age children and negatively affects learning ability and educational potential [10]. There is a gap in understanding why students do not use refractive eye-care services, since no prior studies have examined barriers to the uptake of refractive eye-care services among primary or secondary school children within Uganda.

To address this gap, this study determined the proportion of students with pinhole-improvable reduced distance visual acuity suggestive of uncorrected refractive error and the perceived barriers to using refractive eye care among students from two secondary schools in Kampala, Uganda. Ultimately, this study pinpoints key areas for further investigation and establishes a basis for developing locally adapted school eye-health interventions.

## Materials and methods

### Study design and setting

A cross-sectional study was conducted among students aged 12 years and above from two participating secondary schools, namely Kitante Hill Secondary School, a government-aided school, and Caltec Academy, a private school, between March and April 2019.

### Participants and sampling

The sample size of 407 students was estimated using a simple proportion formula. The following assumptions were used in the calculation: a non-response rate of 10%; the highest proportion of participants reporting a specific barrier limiting uptake of refractive eye-care services, at 61.1% [11]; a 95% confidence interval; and a 5% margin of error. Two participating secondary schools were selected randomly from the 155 secondary schools in Kampala using the lottery method. Two schools were selected due to resource limitations. Study participants were selected through convenience sampling; all students attending the study during the implementation period were screened until the target sample size was reached. This method was used since the study largely focused on a particular subgroup: students with pinhole-improvable reduced distance visual acuity suggestive of uncorrected refractive error (URE), identified during eye screening tests. 800 students were enrolled in the participating government-aided school, and 350 in the participating private school. Students in candidate classes (senior four and senior six) at both schools were unable to participate in the study because they were preparing for mock exams. After excluding students in candidate classes, 790 students were eligible to participate (550 from a government-aided school and 240 from a private school). One week before the screening event, all eligible students were invited to participate through the school administration. Of these, 497 students (300 from a government-aided school and 197 from a private school) picked either parental consent forms or their own participant consent forms. 470 signed consent forms were returned, representing a 94.8% consent form return rate. On the designated screening days, the required sample size of 407 students was screened. The proportion of eligible students screened from a government-aided school was 40.36% (222/550), and from a private school, 77.08% (185/240) (Figure 1).

**Figure 1.**
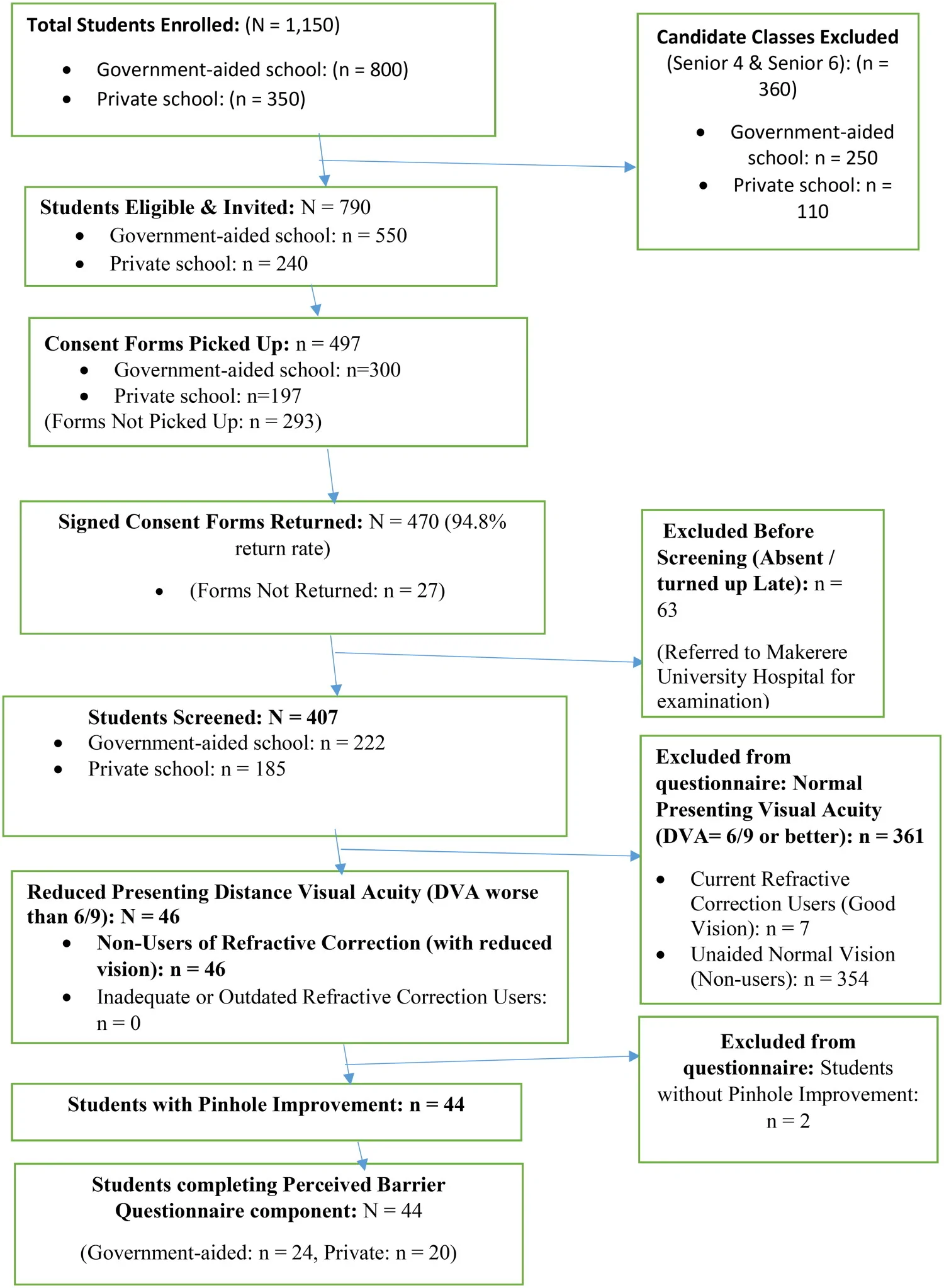
Participant flow diagram.

Some students who returned consent forms did not turn up (63) for the eye screening due to school absence on the eye screening days or showed up after the screening event. Those who missed the eye screening event were advised through the school administration to undergo a comprehensive eye examination at Makerere University Hospital.

Students with pinhole-improvable reduced distance visual acuity suggestive of uncorrected refractive error were assessed to find their perceived barriers to refractive eye-care use.

Students aged 18 years and above who provided consent, as well as students aged 12–17 years who presented a duly signed parental consent form and provided assent, were included in the study. Students aged 12 years and above were included in the study because they had more autonomy in seeking health services than younger children (<12 years of age). Older children also cooperate during vision tests, such as visual acuity. The study excluded students with active ocular pathology, ocular surgery, or abnormal eye conditions, such as nystagmus. These conditions were excluded because they can impair vision. In addition, the study excluded students with known cognitive impairment that could prevent them from providing accurate feedback during eye screening.

### Data collection

Data were collected between March and April 2019 from all participating schools. Data collection took five days at each school, during which presenting distance visual acuity (DVA) was measured under standardized lighting conditions, with overhead and portable light sources adjusted to provide uniform, glare-free chart illuminance between 400 and 600 lux, verified using a handheld digital photometer. Testing was done using a standardized Early Treatment of Diabetic Retinopathy Study (ETDRS) wall chart geometric layout calibrated and printed for a 3-meter testing distance. The testing lane was physically marked on the floor using a measuring tape to ensure an exact 3-meter distance from the subject’s outer eye to the chart surface. The ETDRS VA chart was used because it is a standard research VA chart owing to its higher sensitivity and reliability. Measurements followed a strict monocular protocol, testing the right eye first, then the left, with the contralateral eye occluded using a handheld occluder. DVA results were recorded on visual assessment forms. Those with a DVA worse than 6/9 in either eye were subsequently tested with a pinhole occluder to determine whether the DVA improved. Study participants with unilateral reduced visual acuity (one eye’s DVA worse than 6/9 with a fellow eye’s DVA of 6/9 or better) and bilateral reduced visual acuity (both eyes with DVA worse than 6/9) were combined and analyzed together at the student level. Eye-level findings were converted into student-level classifications based on the status of the eye with poorer vision. A student was classified as having pinhole-improvable reduced visual acuity suggestive of uncorrected refractive error if at least one eye showed improvement in visual acuity during the pinhole test. Standard refraction procedures were not done to ascertain the magnitude and type of refractive error.

Students who were wearing prescribed spectacles or contact lenses were considered to be using refractive eye care services. Students who were wearing spectacles or contact lenses and whose presenting DVA was worse than 6/9 but improved on the pinhole test were considered to have outdated prescriptions or inadequate prescriptions. Direct observation and discussion with the participating students were used to determine whether spectacles or contact lenses were used. Non-utilization of refractive eye-care services was operationally defined as unmet refractive need, specifically presenting with poor vision (distance visual acuity worse than 6/9) that is improvable on the pinhole test, while currently not using any form of refractive correction.

The study participants with pinhole-improvable reduced distance visual acuity suggestive of uncorrected refractive error (all of whom were current non-users of refractive eye-care services) were administered a questionnaire by an interviewer. The questionnaire was adapted from the Andhra Pradesh Eye Disease Study (APEDS) Instrument VIII A (English version), a previously validated instrument used in APEDS (supplementary material 1). There were no pre-validation or language changes to this questionnaire before its use in this study. To minimize potential comprehension challenges during questionnaire administration, trained research assistants were present to provide standardized verbal explanations to students who required explanation of any component of the questionnaire. Permission to utilize the APEDS instrument VIII A in this study was sought from one of the investigators of the APEDS.

Students were required to mark the barrier(s) that limited their uptake of refractive eye-care services. Those whose barriers were not listed were allowed to mention them in the category of “others.” If a student gave more than one response, all responses were recorded for analysis of “any reported barrier.” The student was then asked to identify the single most important barrier. For students who isolated a primary factor, this was recorded as the “most important barrier.” Students who reported multiple concurrent barriers but declined to select a single primary overriding factor, or were unable to do so, were excluded from the primary ranking analysis. A brief interview was also conducted to collect demographic data, including education level, sex, and age of the study participants.

### Data quality control and interviewer training

Data collection was conducted by final-year optometry students pursuing a bachelor’s degree in optometry at Makerere University. All research assistants (RAs) had baseline clinical knowledge of refractive errors. To minimize interviewer-dependent interpretation and ensure consistency across the study participants’ ages, all research assistants underwent a mandatory one-day training workshop. The training focused mainly on standard visual acuity testing and a standardized verbal explanation protocol for the adult-centric questionnaire. RAs were instructed to read questions verbatim and use only the pre-approved, simplified definitions provided in the study manual (Supplementary Material 2) when students sought clarification.

### Data analysis

The collected data were cleaned and subsequently analyzed using Microsoft Office Excel 2010 and Stata (version 10.0; StataCorp, College Station, TX, USA). Descriptive statistics were used to summarize key findings, such as the prevalence of pinhole-improvable reduced distance visual acuity suggestive of URE and the percentage of students who reported a given barrier to uptake of refractive eye-care services. The analyzed data are presented as tables. Multiple responses on barriers to the utilization of refractive eye-care services were evaluated using multiple response frequency analysis, with percentages representing the proportion of total participants (*N* = 44) who selected each specific barrier. Conversely, evaluations of the primary, most important barrier were restricted to the sub-cohort who provided a valid, single response (*N* = 25); participants who did not identify a single primary constraint (*n* = 19) were explicitly treated as missing responses to ensure strict arithmetic reconciliation across questionnaire components. Proportions and their corresponding 95% confidence intervals were calculated using the Wilson score method to accommodate small cell counts. All final values, including point estimates and confidence bounds, were rounded uniformly to two decimal places.

A Chi-square test of independence was initially performed to determine the relationship between sex, age, and pinhole-improvable reduced visual acuity suggestive of URE. The assumptions for the Chi-square tests were verified, as the expected frequencies in some cells were close to zero. To guarantee analytical robustness, Fisher’s Exact Test (using the Fisher–Freeman–Halton extension for multi-row contingency tables) was applied alongside all Chi-square models. Given the limited absolute case volume, all demographic subgroup analyses are designated as exploratory.

### Post hoc conceptual mapping of barriers

To systematically synthesize the identified obstacles to eye care utilization, participants’ responses were mapped post hoc onto the dimensions of the Levesque Client-Centered Framework for Healthcare Access. This framework was not employed prospectively as a baseline measurement instrument during tool design, but rather served as a retrospective interpretive lens for categorizing the empirical data in a secondary analysis.

The classification process followed a rigorous dual-investigator protocol to maximize inter-rater reliability. Two researchers independently assigned each questionnaire barrier item to its primary corresponding Levesque domain. Initial coding assignments were compared, and any discrepancies or classification disagreements between the two primary evaluators were resolved through iterative thematic discussion. A third senior investigator arbitrated unresolved items until a 100.00% consensus was achieved across all categories. It is acknowledged that certain multifaceted, structural barriers inherently possess conceptual overlap and could reasonably span multiple access dimensions. In all such instances of intersection, items were assigned to the single operational domain that represented the primary systemic or economic bottleneck reported by the study participants.

#### Ethical consideration

This study received ethical approval from the Makerere University School of Health Sciences Research and Ethics Committee (approval number: SHREC REF: 2018-071). All procedures were conducted in accordance with the ethical Declaration of Helsinki and its later amendments. Permission was obtained from the Kampala Capital City Authority (KCCA) and the head teachers of the two selected schools three weeks before data collection. The purpose of the study was explained to the participants. All parents/guardians of students who had pinhole-improvable reduced distance visual acuity suggestive of uncorrected refractive error were provided with a summary letter referring them to the Academic Vision Center, Makerere University Hospital, for a comprehensive eye examination. All data collected in this study were backed up and password-protected, with multiple copies kept for future reference.

#### Informed consent

Written informed consent or assent was obtained from the participants or their parents, and assurances of confidentiality were provided. A parental informed consent form was given to each of the students aged 12–17 years to take to their parents. Students aged 12–17 years who returned duly signed consent forms from their parents and provided personal assent were recruited for this study. Students aged 18 years and above provided their own independent written informed consent. Parents of students aged 12–17 years who required more information about the study’s aim or procedures before consenting were asked to come to a specific school on the days of data collection for further clarification from the principal investigator.

## Results

A total of 407 students from two secondary schools participated in this study. More female participants (52.09%) than male participants (47.91%) participated (Table 1). Students had a mean age of 14.31 ± 1.73 years (range: 12–19 years). Most screened participants were aged 12–13 years (40.79%) and 14–16 years (46.44%) (Table 2).

**Table 1.** Distribution of study participants by sex in the selected schools.

Gender	Kitante Hill Secondary School (Government-aided school)	Caltec Academy (Private school)	Total
**Male**	110	85	195
**Female**	112	100	212
**Total**	222	185	407

**Table 2.** Contingency table of distribution of screened participants with pinhole-improvable reduced visual acuity suggestive of uncorrected refractive error by age category.

Age group (years)	Total screened, n(% of Total)	**Students with pinhole-improvable reduced distance visual acuity suggestive of uncorrected refractive error** [Observed (Expected)]	**Students with no pinhole-improvable reduced distance visual acuity suggestive of uncorrected refractive error** [Observed (Expected)]	Odds Ratio (95% CI)	p-value
12–13	166 (40.79%)	18 (17.95)	148 (148.05)	1.00 (Ref)	–
14–16	189 (46.44%)	21 (20.43)	168 (168.57)	1.03 (0.53 to 2.00)	0.932
≥ 17	52 (12.78%)	5 (5.62)	47 (46.38%)	0.87 (0.31 to 2.48)	0.803
TOTAL	407 (100.0%)	44 (44.00)	363 [363.00]	–	0.954

Footnote: Odds Ratios compare each age group to the 12–13 reference group. The overall association was evaluated using the Chi-square test (X^2^ = 0.095, *p* = 0.954). Fisher-Freeman-Halton Exact Test p-value: *p* = 0.961.

### Proportion of screened students with pinhole-improvable reduced distance visual acuity suggestive of uncorrected refractive error

A total of 46 students presented with distance visual acuity worse than 6/9 (reduced distance visual acuity) in one or both eyes. When evaluated by the pattern of eye involvement, 10 students (10/46, 21.74%) presented with unilateral reduced DVA (DVA worse than 6/9 in the poorer eye with a fellow eye of 6/9 or better), while 36 participants (78.26%) presented with bilateral reduced DVA (both eyes worse than 6/9). On the pinhole test, 44 students had pinhole-improvable reduced distance visual acuity suggestive of uncorrected refractive error (URE), representing 10.81% of the screened students (44/407, 95% CI: 8.15% to 14.20%). Crucially, 100% (44/44) of these identified students were non-users of spectacles or contact lenses on the screening day; none of the current users met the inclusion criteria for the final cohort. Conversely, 2 students with reduced presenting DVA did not show visual improvement on the pinhole test (Table 3).

**Table 3.** Showing visual acuity and pinhole response profile by laterality of reduced distance vision (*N* = 46).

Ocular Metric	Unilateral Reduced DVA (*n* = 10)	Bilateral Reduced DVA (*n* = 36)	Total Cohort (*N* = 46)
Pattern of eye involvement	10 (21.74%)	36 (78.26%)	46 (100.00%)
**Presenting distance visual acuity (DVA): Poorer Eye**	**Presenting distance visual acuity (DVA): Poorer Eye**		
Worse than 6/9	10 (100.0%)	36 (100.0%)	46 (100.00%)
**Presenting DVA: Better Eye**			
6/9 or Better	10 (100.0%)	0 (0.0%)	10 (21.74%)
Worse than 6/9	0 (0.0%)	36 (100.00%)	36 (78.26%)
**Pinhole Response Profile**			
Pinhole Improvable (One or Both Eyes)	10(100.00%)	34(94.44%)	44 (95.65%)
No Pinhole Improvement	0(0.00%)	2(5.56%)	2 (4.35%)

Exploratory analyses of pinhole-improvable reduced distance visual acuity distribution across age and sex subgroups revealed no statistically significant associations. Pinhole-improvable reduced distance visual acuity suggestive of uncorrected refractive error was found in 12.74% (27/212) of females, compared with 8.72% (17/195) of males. This variance did not reach statistical significance (X^2^ = 1.31, *p* = 0.253; Fisher’s exact *p* = 0.205; OR = 1.53, 95% CI: 0.81 to 2.90). Minimum expected cell counts for the gender matrix were verified at 21.08 (Table 4).

**Table 4.** Contingency table of distribution of screened participants with pinhole-improvable reduced distance visual acuity suggestive of uncorrected refractive error by sex.

Gender	Screened participants with pinhole-improvable reduced distance visual acuity suggestive of uncorrected refractive error [Observed (Expected)]	Screened participants with no pinhole-improvable reduced distance visual acuity suggestive of uncorrected refractive error [Observed (Expected)]	Total Screened
Female	27 (22.92)	185 (189.08)	212
Male	17 (21.08)	178 (173.92)	195
Total	44	363	407

Footnote: Pearson Chi-square: χ^2^ = 1.31, *p* = 0.253, Fisher’s Exact Test p-value: *p* = 0.205.

The distribution of students with pinhole-improvable reduced distance visual acuity suggestive of uncorrected refractive error in each age category was found to be 10.84% (18/166) among 12–13-year-old students, 11.11% (21/189) among 14–16-year-old students, and 9.62% (5/52) among students >17 years (Table 2). The minimum expected cell count for the age model was calculated to be 5.62 in the ≥17-year cohort, consistent with standard assumptions (Table 2). Using age category 12–13 years as a reference, the odds of having URE did not significantly differ for the 14–16 years’ age group (OR = 1.03, 95% CI: 0.53 to 2.00) or the ≥ 17 years’ age group (OR = 0.87, 95% CI: 0.31 to 2.48). This study found no statistically significant relationship between age and pinhole-improvable reduced distance visual acuity suggestive of URE (χ^2^ = 0.0949, *p* = 0.9536). The extended multi-row exact model confirmed the lack of association (Fisher–Freeman–Halton exact *p* = 0.961).

### Perceived barriers to uptake of refractive eye-care services among current spectacle/contact lens non-users

Regarding any reported barrier (multiple responses allowed), 56.82% (25/44) of student indicated that more than one barrier obstructed their uptake of refractive eye-care services. The perceived barriers reported included fear of surgery (23/44, 52.27%, 95% CI: 37.94% to 66.25%), not knowing where to go for an eye checkup (22/44, 50%, 95% CI: 35.83% to 64.17%), not feeling comfortable with the indigenous practitioner to whom they had access (19/44, 43.18%, 95% CI: 29.68% to 57.78%) and being afraid that seeing someone for an eye checkup could reveal vision loss and, thereafter, cause worry (18/44, 40.91%, 95% CI: 27.69% to 55.59%). Lack of money to cover treatment expenses (18/44, 40.91%, 95% CI: 27.69% to 55.59%) was also reported as a perceived barrier (Table 5).

**Table 5.** Frequency and prioritization of perceived barriers to the uptake of refractive eye care services among screened students with pinhole-improvable reduced distance visual acuity suggestive of uncorrected refractive error (*N* = 44).

Barrier description	Any barrier (*N* = 44, multiple choices allowed) n (%) [95% CI]	Most important barrier (*N* = 25, single choice allowed) n (%) [95% CI]
Fear of surgery	23 (52.27%) [37.94% to 66.25%]	—
Don’t know where to go for an eye checkup	22 (50.00%) [35.83% to 64.17%]	2 (8.00%) [2.20% to 25.00%]
Uncomfortable with the indigenous practitioner	19 (43.18%) [29.68% to 57.78%]	—
Afraid of revealing vision loss, which therefore causes worry	18 (40.91%) [27.69% to 55.59%]	2 (8.00%) [2.20% to 25.00%]
No money to meet treatment expenses	18 (40.91%) [27.69% to 55.59%]	1 (4.00%) [0.70% to 19.50%]
Have to travel far for an eye checkup	16 (36.36%) [23.78% to 51.13%]	—
No money to pay for an eye checkup	15 (34.09%) [21.88% to 48.86%]	5 (20.00%) [8.90% to 39.10%]
Uncomfortable with the homeopathic practitioner	13 (29.55%) [18.16% to 44.22%]	—
Uncomfortable with the eye doctor	13 (29.55%) [18.16% to 44.22%]	—
Other obligations prevent an eye checkup	12 (27.27%) [16.35% to 41.85%]	3 (12.00%) [4.20% to 30.00%]
Loss of wages–self	12 (27.27%) [16.35% to 41.85%]	—
No money to travel	10 (22.73%) [12.84% to 36.99%]	1 (4.00%) [0.70% to 19.50%]
Not a priority because of other serious medical problems	7 (15.91%) [7.93% to 29.37%]	—
Loss of wages of accompanying person	7 (15.91%) [7.93% to 29.37%]	—
Able to see adequately	6 (13.64%) [6.40% to 26.71%]	2 (8.00%) [2.20% to 25.00%]
Other medical problems prevent me	6 (13.64%) [6.40% to 26.71%]	—
Dissatisfaction of another person with treatment	6 (13.64%) [6.40% to 26.71%]	—
Nobody to look after me after surgery	4 (9.09%) [3.59% to 21.16%]	—
Others *	2 (4.55%) [1.26% to 15.13%]	—
Dissatisfaction of another person with the hospital	2 (4.55%) [1.26% to 15.13%]	—
The dominant person did not feel the need.	—	3 (12.00%) [4.20% to 30.0%]
No serious problem	—	4 (16.00%) [6.40% to 34.70%]
A decrease in vision is natural when growing old	—	2 (8.00%) [2.20% to 25.00%]
Missing / No single primary barrier specified	—	19 (43.18%)
Total Valid Responses	—	25 (100.00%)

95% Confidence Interval (calculated *via* Wilson Score method). Percentages in column 1 do not sum to 100% because multiple responses were permitted. Percentages in column 2 sum to 100% among the *N* = 25 who identified a single most important barrier; the remaining 19 participants did not select a single primary barrier. Responses under “Others” included: “spectacles make eyes smaller” and “spectacles kill eyes gradually.

However, several barriers were encountered when cell counts were very small (*n* ≤ 7), yielding highly unstable point estimates and noticeably wide 95% confidence intervals that require extreme caution. For instance, prioritizing other medical problems (15.9%, 95% CI: 7.9%–29.4%), lacking a post-surgical caregiver (9.1%, 95% CI: 3.6%–21.2%), and hospital dissatisfaction (4.5%, 95% CI: 1.3%–15.1%) demonstrated wide intervals due to low statistical power. Although these low point estimates suggest these barriers are less dominant in the sample, the underlying population proportion remains highly uncertain because the URE subgroup has a limited sample size (*N* = 44).

### The prioritization of the most important perceived barrier to refractive services

When students with pinhole-improvable reduced distance visual acuity suggestive of uncorrected refractive error were asked to identify the single most important barrier, only 25 students did so. They indicated lack of money to pay for eye tests as their most important barrier for refractive eye-care service uptake (5/25, 20.00%, 95% CI:95% CI: 8.86% to 39.13%), followed because of the belief that they had no serious eye problems (4/25, 16.00%, 95% CI: 6.40% to 34.65%), having the dominant person in the family not feeling the need to seek eye care (3/25, 12.00%, 95% CI: 4.17% to 29.96%) and other obligations preventing an eye checkup (3/25, 12.00%, 95% CI: 4.17% to 29.96%). Additional prioritized barriers reported by 8.00% (2/25, 95% CI: 2.22% to 24.97% each) of this sub-cohort included the ability to see adequately, the belief that a decrease in vision is natural when growing old, fear of revealing vision loss, and not knowing where to go for an eye checkup. 43.18% of the participants with pinhole-improvable distance visual acuity (19/44, 95% CI: 29.68% to 57.78%) did not state any particular barrier as their most important (Table 5).

Barriers not stated as most important included having to travel long distances for an eye checkup, no one willing to escort them for an eye checkup, and not prioritizing an eye checkup because of other medical conditions. Additionally, no participant indicated feeling uncomfortable with the eye care providers they had access to, being apprehensive because of dissatisfaction with another person’s treatment, dissatisfaction with the hospital, or fear of surgery as the most important barrier to uptake of refractive services.

## Discussion

### Proportion of screened students with pinhole-improvable reduced distance visual acuity suggestive of uncorrected refractive error

In total, 10.81% (44/407) of the students had pinhole-improvable reduced distance visual acuity suggestive of uncorrected refractive error (URE); of these, 61.36% (27/44) were female and 38.64% (17/44) were male. This study’s finding is considerably lower than the 27.1% URE prevalence found in a cross-sectional study conducted among high school students in South Africa^5^ ,yet higher than the prevalence obtained from similar school-based studies done in Bongo district in Ghana, Kakamega in Kenya, and Zimbabwe [12–14]. However, these cross-sectional comparisons should be interpreted with caution due to differences in the outcome definitions across studies. While this study relied on pinhole-improvable reduced distance acuity to identify students with potential URE, many reference studies used cycloplegic refraction. A non-cycloplegic pinhole test may underestimate the prevalence of URE, particularly uncorrected latent hyperopia, because children and adolescents have a high accommodative amplitude.

In this study, we identified several self-reported barriers prevalent among screened students with pinhole-improvable reduced distance visual acuity suggestive of uncorrected refractive error, which may co-occur with lower uptake of refractive eye-care services. These included the cost of refractive eye care services, fear of surgery, not knowing where to go for an eye checkup, and fear that an eye checkup could reveal vision loss. While the descriptive cross-sectional study design utilized in this study does not establish a causal relationship between barriers and the observed proportion of pinhole-improvable reduced distance visual acuity suggestive of URE, these factors are often associated with poor utilization of refractive services.

### Perceived barriers to refractive eye-care service uptake

The perceived barriers to refractive eye care use among secondary school students who participated in the study are multifaceted and dynamically interconnected. To synthesize these findings, a multidimensional access framework, adapted from the Levesque Conceptual Framework for Healthcare Access, was applied post hoc as an interpretive organizational lens to categorize the perceived barriers (Table 6). In this study, dimensions related to the “Ability to Perceive” (knowledge and attitudes) were prominent barriers to using refractive eye care services. A significant proportion of students held misconceptions that they were “able to see adequately” and believed that a decrease in vision is simply a natural consequence of growing older, which might have masked their underlying uncorrected refractive errors. In addition, 50.00% reported that they “did not know where to go for an eye checkup” when they suspected they had vision issues.

**Table 6.** A table showing the category of each barrier to refractive eye-care service uptake.

	BARRIER categorization
1.	**Knowledge and attitudes** Don’t know where to go for an eye checkup Able to see adequately A decrease in vision is natural when growing old
2.	**Family and Social Support** The dominant person did not feel the need Loss of wages of accompanying person Nobody to look after me after surgery
3.	**Affordability** No money to meet treatment expenses No money to pay for an eye checkup No money to travel Loss of wages (self)
4.	**Geographic Accessibility** Have to travel far for an eye checkup
5.	**Service Acceptability** Fear of surgery Uncomfortable with the indigenous practitioner/eye doctor/homeopath Dissatisfaction with another person with treatment/hospital
6.	**Health-System/Contextual Factors** Other obligations prevent an eye checkup Not a priority because of other serious medical problems

Importantly, the lack of knowledge was compounded by service acceptability factors (mapping to the “Ability to Seek” domain), particularly a high selection rate for “fear of surgery”(52.27%) and varying levels of discomfort with eye care providers. This might reflect a significant breakdown in trust and communication within the local eye care infrastructure. When people lack clear information about eye care services, anxieties can become insurmountable barriers to seeking care. Targeted outreach campaigns to improve knowledge and elicit changes in attitudes and behaviors among students, teachers, and parents should be implemented in participating schools to ensure the effective use of refractive services.

Concerning the healthcare supply side, affordability appeared as a cross-cutting structural constraint affecting the **“**Ability to Pay.” It is worth noting that certain multifaceted barriers, such as travel expenses and the loss of wages, exhibit conceptual overlap across both affordability and geographic accessibility domains; for our analytical mapping, these were classified under the domain representing the primary financial bottleneck. Interestingly, while some participants reported geographic accessibility (“has to travel far”), its impact as a primary barrier is relatively low. This finding is consistent with the rapidly changing eye care environment in Kampala and Central Uganda, where the geographical availability of refractive services has expanded significantly. The private sector has largely driven the growth through the establishment of several private eye clinics and optical shops, as well as the efforts of the National Intervention on Uncorrected Refractive Error (NIURE) program (2008–2020) [15]. Affordability concerns are likely due to the fact that refractive services are not yet fully integrated into Uganda’s healthcare system; that is, most public health facilities in Uganda do not provide them. Private eye care practices primarily provide refractive services; therefore, students who cannot afford private fees cannot obtain them from government-run health facilities at a subsidized cost or for free.

This post hoc grouping also stresses the key role of family and social support in adolescent eye health. Barriers such as a “dominant person in the family not feeling the need” for care, the “loss of wages of an accompanying person,” or having “nobody to look after me after surgery” reflect that adolescent refractive eye care utilization is rarely an autonomous decision. Finally, these familial factors are constrained by wider health-system and contextual factors such as “other serious medical problems” or “other obligations.”

While our study did not prospectively measure the Levesque constructs using a structured, framework-based questionnaire, mapping our empirical data onto these dimensions allows our findings to contribute to the existing regional literature. A cross-sectional study conducted in South India by Marmamula and colleagues concluded that knowledge, attitude, and acceptability were the most commonly cited barriers to the uptake of refractive services [11]. Conversely, a similar study in Mozambique identified only supply-side structural barriers, with 53% of participants reporting “difficulty paying the cost of services” (affordability) and 15% reporting “distance to the services” (geographic accessibility) as their main limitations [16].

In this study, 52.27% of students with pinhole-improvable reduced distance visual acuity cited fear of surgery as their perceived barrier to uptake of refractive eye care services. This may reflect misinterpretation of the questionnaire or misunderstanding of eye care services. This misunderstanding can create a psychological barrier, even when the services are easily available. Refractive errors in children and teenagers are usually managed with non-invasive spectacles or contact lenses rather than surgery.

Furthermore, data collection occurred between March and April 2019. While these findings provide a vital baseline towards comprehending perceived barriers among secondary school students in Kampala, the structure, availability, and affordability of refractive services may have changed over the years. Consequently, statements about private sector dominance, geographic expansion, or the lack of public health infrastructure coordination should be interpreted as indicating the service environment at the time of data collection rather than a definitive portrait of present-day service access.

Lastly, while the number of screened students was robust (*N* = 407), the proportion of screened participants with pinhole-improvable reduced distance visual acuity suggestive of URE was relatively small (*N* = 44). Therefore, treat all comparative assessments across sex and age as exploratory findings. Furthermore, because the subsequent barrier analysis was restricted to this small subgroup, the effective sample sizes (*N* = 44 for any barriers and *N* = 25 for the most important barrier) limit statistical precision. Therefore, interpret individual barrier percentages and their wide confidence intervals with caution, as exploratory. Notably, the reported confidence intervals do not account for school-level clustering. Because data collection was restricted to two schools, the study could not reliably estimate cluster-level variance. These confidence intervals must therefore be interpreted as strictly descriptive of the participating sample rather than as precise population-level parameters for all secondary school students across Kampala.

#### Strengths and limitations of the study

The main strength of this study is its targeted inclusion criteria: only students with pinhole-improvable reduced distance visual acuity suggestive of URE were required to identify perceived barriers to utilization of refractive eye care services. The study’s limitations comprise **t**he use of convenience sampling to recruit participants, which could have introduced selection bias; therefore, the results should be generalized with caution to all students in Kampala. In addition, the study was performed in only two schools due to funding limitations. The findings may be subject to interviewer and social desirability bias because of the adolescent cohort and the questionnaire’s face-to-face administration. The interactive nature of the verbal explanations of the questionnaire might have influenced how some students perceived the questions.

Furthermore, adolescent students may have over- or underreported specific barriers to match their anticipated expectations of the Interviewer. In addition, the study did not formally record individual participants’ comprehension difficulties, as the questionnaire was not originally designed for adolescents and was not piloted among Ugandan adolescents. Some students might have failed to contextualize certain adult-centric terms in the questionnaire, despite the continuous presence of trained research assistants who provided standardized verbal clarification. The contextual detachment likely led to a high selection rate for “fear of surgery” (52.27%) as a barrier to uptake of refractive eye-care services; participants frequently conflated non-invasive refractive services with invasive surgical eye care. The pinhole testing utilized in the study does not specify the type and magnitude of refractive errors. Additional research should use a larger sample size and random sampling techniques to reduce selection bias and produce more generalizable findings.

Furthermore, the wide age range and diverse school environments (government-aided and private schools) among students likely introduced measurement error and variation in how participants interpreted the questions. Younger students (aged 12–14) may have lacked the health literacy to distinguish between routine non-invasive refractive care and invasive ocular surgery, which could have led to a higher selection rate for the ‘fear of surgery’ option. Similarly, abstract economic questions about ‘loss of wages’ or unfamiliar terms such as ‘Unani practitioners’ were likely outside the lived experience of younger adolescents compared with older participants (aged 17–19).

Differences in school types across Kampala may have also introduced interpretation bias. Students attending government-aided schools may have been more sensitive to financial terms, interpreting ‘cost barriers’ through the lens of household monetary constraints. In contrast, students from high-resource private schools might have interpreted these items differently. Additionally, the proportion of eligible students who were screened differed substantially between the government-aided school (40.36%) and the private school (77.08%). This difference might have influenced both the observed proportion of pinhole-improvable reduced distance visual acuity and the reported barrier profile.

Because data collection relied on verbal clarifications from the research assistants to bridge these comprehension gaps, minor interviewer-dependent variations in interpretation throughout age brackets and schools might have occurred. The requirement for written parental consent may have introduced selection bias. Parents with greater awareness of refractive errors or refractive eye care, or those who noticed vision problems in their children, may have been more likely to consent. Consequently, our sample might have overrepresented students with perceived eye issues or families already inclined to seek eye healthcare. In addition, selection bias might have come from school absenteeism on the days of data collection. Students absent during the screening might differ systematically in socioeconomic status or eye health-seeking behavior from those present, possibly biasing the perceived barriers reported.

Furthermore, self-perceived visual difficulty may have affected participation. Students with significant visual discomfort may have been highly motivated to participate to receive a free eye checkup. Conversely, those who are asymptomatic or unaware of their reduced visual acuity may have lacked the motivation to return consent forms, underestimating their need for refractive services. In addition, excluding 360 students in senior four and senior six due to intense mock-examination preparation limits the generalizability of our findings. Finally, we acknowledge that this study was not specifically powered to estimate individual barriers with high precision.

## Supplementary Material

QUESTIONAIRE.docx

EXPLANATIONS FOR ADULT CENTRIC QUESTIONS.docx

## Data Availability

The datasets obtained during the study are available from the corresponding author upon request.

## References

[1] Steinmetz JD, Bourne RRA, Briant PS, et al. Global estimates on the number of people blind or visually impaired by uncorrected refractive error: a meta-analysis from. Eye (Lond). 2024;38(6):2083–2101. 10.1038/s41433-024-03106-0 PMC11269735 38965322

[2] World Health Organization. Report of the 2030 Targets on Effective Coverage of Eye Care. World Health Organization; 2022.

[3] Horton S, Gelband H, Jamison D, et al. Ranking 93 health interventions for low- and middle-income countries by cost-effectiveness. PLoS One. 2017;12(8):e0182951. doi: 10.1371/journal.pone.0182951. PMC5552255 28797115

[4] Srivastava T, Kumar A, Shukla E, et al. Prevalence of refractive errors among school-going children in urban versus rural areas. Cureus. 2024;16(4):e59197. doi: 10.7759/cureus.59197. PMC11131348 38807816

[5] Nyathela X, Nirghin U, Ebrahim Khan N. Prevalence of uncorrected refractive error in low-resource high schools in the Free State, South Africa. Afr J Prim Health Care Fam Med. 2025;17(1):e1–e8. doi: 10.4102/phcfm.v17i1.4967. PMC12506818 41055186

[6] Kawuma M, Mayeku R. A survey of the prevalence of refractive errors among children in lower primary schools in Kampala district. Afr Health Sci. 2002;2(2):69–72. PMC2141567 12789105

[7] Kamara D, Onyango J, Arunga S, et al. Prevalence and factors associated with refractive errors among university students at Mbarara University of Science and Technology, Uganda. OJOph. 2024;14(03):324–338. doi: 10.4236/ojoph.2024.143030.

[8] Saad A, El-Bayoumy B. Environmental risk factors for refractive error among Egyptian schoolchildren. East Mediterr Health J. 2007;13(4):819–828. 17955764

[9] Uganda Radio Network. Government raises alarm over soaring eye problems among Ugandan children. The Observer. 2025 Nov 13 [cited on 21st July 2026]. Available from: https://observer.ug/news/govt-raises-alarm-over-soaring-eye-problems-among-ugandan-children/.

[10] Resnikoff S, Pascolini D, Mariotti SPM, et al. Global magnitude of visual impairment caused by uncorrected refractive errors in 2004. Bull World Health Organ. 2008;86(1):63–70. doi: 10.2471/blt.07.041210. PMC2647357 18235892

[11] Marmamula S, Keefe JE, Ramaan U, et al. A population-based cross-sectional study of the barriers to utilization of refractive services in South India: the Andhra Pradesh Eye Disease Study. BMJ Open. 2011;1(2):e000172. 10.1136/bmjopen-2011-000172 PMC3191577 22021782

[12] Asare FA, Morjaria P. Prevalence and distribution of uncorrected refractive error among school children in the Bongo District of Ghana. Cogent Med. 2021;8(1):1911414. doi: 10.1080/2331205X.2021.1911414.

[13] Okenwa-Vincent EE, Naidoo J, Clarke-Farr P. Prevalence and socio-demographic distribution of uncorrected refractive errors in school-going adolescents in Kakamega County, Kenya. Ophthalmol J. 2024;9(0):45–52. doi: 10.5603/oj.96882.

[14] Lou L, Yao C, Jin Y, et al. Global patterns in health burden of uncorrected refractive error. Invest Ophthalmol Vis Sci. 2016;57(14):6271–6277. doi: 10.1167/iovs.16-20242. 27893092

[15] Mullegger B. Learning Guide: NIURE. Light for the World; 2014. [cited 2025 Jan 30]. Available from: https://www.light-for-the-world.org

[16] Thompson S, Naidoo K, Gonzalez-Alvarez C, et al. Barriers to use of refractive services in Mozambique. Optom Vis Sci. 2015;92(1):59–69. doi: 10.1097/OPX.0000000000000431. PMC4274338 25379630

[17] Levesque JF, Harris MF, Russell G. Patient-centered access to health care: conceptualizing access at the interface of health systems and populations. Int J Equity Health. 2013;12(1):18. doi: 10.1186/1475-9276-12-18. PMC3610159 23496984

