# Supplementary material for: Perceived barriers to eye care use among secondary school students with pinhole-improvable reduced visual acuity in two schools in Kampala, Uganda: a cross-sectional study
Source: Ann Med. 2026 Oct 5;58(1):2743336. doi: 10.1080/07853890.2026.2743336 (PMC13644455; doi:10.1080/07853890.2026.2743336)
Supplement: Supplementary file 1 [file IANN_A_2743336_SM0943.docx]

**SUPPLEMENTARY MATERIAL 2: STANDARDISED VERBAL EXPLANATION PROTOCOL**

**INSTRUCTIONS FOR RESEARCH ASSISTANTS(RAs):**

- Do **not** read these explanations out loud by default.
- Keep a neutral tone. Do not suggest or prompt answers.
- Only read an explanation **if a student asks for clarity** or shows confusion about a specific question.

**PART 1: EYE CARE PROVIDER ROLES (QUESTIONS 803A.5 & 803A.6)**

Students often confuse different eye professionals. Use these definitions:

- **1. Eye Doctor / 2. Ophthalmologist / 3. Optometrist / 4. Optician:** "These are the specialists who test eyes, prescribe glasses, or do eye operations. If you saw a professional at an eye clinic or eye hospital, it falls here."
- **5. General Medical Doctor:** "This is a regular doctor or clinical officer at a general hospital, general clinic, or health center who treats general sicknesses like malaria or cough, not an eye specialist."
- **6. Homeopathic / Ayurvedic / Unani Practitioner (from 805A.12):** "These are practitioners who use alternative, non-Western natural medicines or herbal tablets, often from Indian or international natural health systems."
- **7. Indigenous Practitioner:** "This refers to local traditional herbalists or community healers who use local herbs and traditional methods to treat illnesses."
- **8. Spiritual Healers:** "This refers to pastors, prayer warriors, religious leaders, or shrine leaders whom people visit to pray over their health conditions."

**PART 2: SECTION 805A - BARRIERS TO REFRACTIVE EYE CARE**

Use these simplified explanations to make clear complex adult phrases:

- **805A.22 & 805A.23. Apprehensive because of dissatisfaction of another person...**
- **805A.1. Dominant person in family, if other than the subject, did not feel the need for seeking health care**
- Explanation: "This means your parent, guardian, or the person who pays the bills at home thinks your refractive eye care is small and decided you do not need to go to a clinic/hospital."
- **805A.4. Eye disease/decrease in vision are natural with growing old and so don’t need treatment**
- Explanation: "This means believing that blurry vision is just a normal thing that happens as people grow up or get older, so there is no point in trying to fix it."
- **805A.5. Afraid that seeing someone for an eye check-up would reveal vision loss, and therefore cause worry**
- Explanation: "This means you are scared that if the doctor tests you, they will tell you that your eyes are badly damaged, and that bad news will stress or frighten you."
- **805A.9. Family/harvest/business/other obligations prevent eye check-up.**
- Explanation: "This means you are too busy with household chores, helping your parents run their business/shop, looking after siblings, or schoolwork to find time to go to the hospital."
- **805A.11 to 805A.13. Do not feel comfortable with the practitioner...**
- Explanation: "This means you do not trust or feel safe with the doctors or local healers available to you, or you are afraid they might mistreat you."
- **805A.14 & 805A.15. Eye check-up not a priority because of other medical problems…**
- Explanation: "This means you or your family are managing a much more severe illness right now, so you are spending all your attention and money on that instead of your eyes."
- **805A.17. Nobody to look after me after surgery**
- Explanation: "This means you are worried that if you get a major eye procedure, there will be no one at home or school to cook for you, help you move around, or take care of you while you heal."
- **805A.20 & 805A.21. Loss of wages (Self / Accompanying person) ...**
- Explanation: "This means that taking a day off to go to the eye clinic causes your parents to lose money because they have to close their business or skip work for that day."
- Explanation: "This means you are hesitant or afraid to go because a friend, classmate, or family member went to that clinic or got eye treatment before and had a terrible experience.
- **805A.24. Fear of surgery**
- Explanation for Student: "This means you are deeply afraid or worried about the idea of an eye operation, needles, or medical tools touching your eyes, so you avoid going for a check-up altogether to prevent that from happening.
