# Supplementary material for: Perceived barriers to eye care use among secondary school students with pinhole-improvable reduced visual acuity in two schools in Kampala, Uganda: a cross-sectional study
Source: Ann Med. 2026 Oct 5;58(1):2743336. doi: 10.1080/07853890.2026.2743336 (PMC13644455; doi:10.1080/07853890.2026.2743336)
Supplement: Supplementary file 2 [file IANN_A_2743336_SM0945.docx]

**SUPPLEMENTARY MATERIAL 1: QUESTIONNAIRE**

STUDY ID NUMBER………………………….CLASS………………………GENDER……….

DATE OF INTERVIEW……………………………….

ADMINISTERED TO STUDENTS ≥12 YEARS OLD ONLY	Applicable/not applicable

| QUESTIONS | RESPONSE |
| --- | --- |
| 803A.1.HAVE YOU NOTICED A CHANGE IN YOUR VISION?  0 No  1Yes  98 can’t say |  |
| 803A.2.HAVE YOU SOUGHT TREATMENT FOR IT?  0 No go to 505A  1. Yes |  |
| 803A.3.DID YOU SEEK CARE FROM MORE THAN ONE EYE CARE PROVIDER FOR THIS EPISODE?  0 no go to 803A.6  1 yes |  |
| 803A.4.DID YOU SEEK CARE FROM MORE THAN ONE EYE CARE PROVIDER BECAUSE OF DISSATISFACTION WITH THE PREVIOUS ONE[S]?  [0.no ,1.YES] If “0” go to 803A.6 |  |
| 803A.5. IF YOU SOUGHT MORE THAN ONE EYE CARE PROVIDER BECAUSE OF DISSATISFACTION WITH THE PREVIOUS ONE[S],  SPECIFY THE PERSON YOU ARE DISSATISFIED WITH.  1. Eye doctor 6. Homeopathic/ayurvedic  2. Ophthalmologist 7. indigenous practitioner  3. Optometrist 8. Spiritual healers  4. Optician 9. Others  5. General medical doctor 10. don't know  [If 803A.4 is 0 record this as -98, not applicable]  . |  |
| 803A.6. WHO DID YOU SEE LAST FOR THIS EPISODE?  1. Eye doctor 6. Homeopathic /ayurvedic  2. Ophthalmologist 7. Indigenous practitioner  3. Optometrist 8. Spiritual healers  4. Optician 9. Others  5. General medical doctor 10. Don’t know |  |
| 803A.7.HOW MUCH WERE YOU SATISFIED WITH THIS VISIT  0. NOT AT ALL  1. SLIGHTLY  3. SOMEWHAT  4. VERY SATISFIED |  |
| 803A.8. HOW MUCH WERE YOU SATISFIED WITH THE FINAL RESULTS? 0. NOT AT ALL  1. SLIGHTLY  3. SOMEWHAT  4. VERY SATISFIED |  |

**805A.BARRIERS TO REFRACTIVE EYE CARE USE**

| WHY HAVE YOU NOT SEEN ANYONE FOR YOUR EYE CHECK?  SPECIFY REASONS | RESPONSE  0. No  1. Yes  2. Most important |
| --- | --- |
| 805A.1. DOMINANT PERSON IN FAMILY, IF OTHER THAN THE SUBJECT, DID NOT FEEL THE NEED TO SEEK HEALTH CARE |  |
| 805A.2. DON'T HAVE A SERIOUS PROBLEM |  |
| 805A.3. ABLE TO SEE ADEQUATELY |  |
| 805A.4. EYE DISEASE/DECREASE IN VISION ARE NATURAL WITH GROWING OLD AND SO DON’T NEED TREATMENT |  |
| 805A.5. AFRAID THAT SEEING SOMEONE FOR AN EYE CHECK-UP WOULD REVEAL VISION LOSS, AND THEREFORE CAUSE WORRY |  |
| 805A.6. DON'T KNOW WHERE TO GO FOR EYE CHECK-UP |  |
| 805A.7. HAVE TO TRAVEL FAR FOR EYE CHECK-UP |  |
| 805A.8. NO ONE IS WILLING TO ESCORT FOR EYE CHECK-UP |  |
| 805A.9. FAMILY/HARVEST/BUSINESS/OTHER OBLIGATIONS PREVENT EYE CHECK-UP |  |
| 805A.10. DON'T HAVE THE MONEY TO PAY FOR EYE CHECK-UP |  |
| 805A.11. DON'T FEEL COMFORTABLE WITH THE INDIGENOUS PRACTITIONER THAT I HAVE ACCESS TO |  |
| 805A.12. DON’T FEEL COMFORTABLE WITH THE HOMEOPATHIC/UNANI PRACTITIONER THAT I HAVE ACCESS TO |  |
| 805A.13. DO NOT FEEL COMFORTABLE WITH THE EYE DOCTOR/GENERAL MEDICAL DOCTOR WHOM I HAVE ACCESS TO |  |
| 805A.14. EYE CHECK-UP NOT A PRIORITY BECAUSE OF OTHER SERIOUS MEDICAL PROBLEMS |  |
| 805A.15.WOULD LIKE EYE CHECK-UP BUT OTHER MEDICAL PROBLEMS PREVENT ME FROM GOING |  |
| 805A.16. OTHERS[SPECIFY] |  |
| 805A.17. NOBODY TO LOOK AFTER ME AFTER SURGERY |  |
| 805A.18. NO MONEY TO TRAVEL TO EYE HOSPITAL |  |
| 805A.19. NO MONEY TO MEET TREATMENT EXPENSES |  |
| 805A.20. LOSS OF WAGES-SELF |  |
| 805A.21. LOSS OF WAGES-ACCOMPANYING PERSON. |  |
| 805A.22. APPREHENSIVE BECAUSE OF DISSATISFACTION OF ANOTHER PERSON WITH TREATMENT |  |
| 805A.23. APPREHENSIVE BECAUSE OF DISSATISFACTION OF ANOTHER PERSON WITH HOSPITAL |  |
| 805A.24. FEAR OF SURGERY |  |
